# Food mechanical properties and isotopic signatures in forest versus savannah dwelling eastern chimpanzees

**DOI:** 10.1038/s42003-018-0115-6

**Published:** 2018-08-10

**Authors:** Adam van Casteren, Vicky M. Oelze, Samuel Angedakin, Ammie K. Kalan, Mohamed Kambi, Christophe Boesch, Hjalmar S. Kühl, Kevin E. Langergraber, Alexander K. Piel, Fiona A. Stewart, Kornelius Kupczik

**Affiliations:** 1 Max Planck Weizmann Center for Integrative Archaeology and Anthropology, Max Planck Institute for Evolutionary Anthropology, Deutscher Platz 6, 04103 Leipzig, Germany; 2 Anthropology Department, University of California Santa Cruz, 1156 High Street, Santa Cruz, CA 95064 USA; 3 Department of Primatology, Max Planck Institute for Evolutionary Anthropology, Deutscher Platz 6, 04103 Leipzig, Germany; 4 German Centre for Integrative Biodiversity Research (iDiv), Halle-Jena-Leipzig, Deutscher Platz 5e, 04103 Leipzig, Germany; 5 School of Human Evolution and Social Change and Institute of Human Origins, Arizona State University, Tempe, AZ 85281 USA; 6 School of Natural Sciences and Psychology, Liverpool John Moores University, Liverpool, L3 3AF UK

## Abstract

Chimpanzees are traditionally described as ripe fruit specialists with large incisors but relatively small postcanine teeth, adhering to a somewhat narrow dietary niche. Field observations and isotopic analyses suggest that environmental conditions greatly affect habitat resource utilisation by chimpanzee populations. Here we combine measures of dietary mechanics with stable isotope signatures from eastern chimpanzees living in tropical forest (Ngogo, Uganda) and savannah woodland (Issa Valley, Tanzania). We show that foods at Issa can present a considerable mechanical challenge, most saliently in the external tissues of savannah woodland plants compared to their tropical forest equivalents. This pattern is concurrent with different isotopic signatures between sites. These findings demonstrate that chimpanzee foods in some habitats are mechanically more demanding than previously thought, elucidating the broader evolutionary constraints acting on chimpanzee dental morphology. Similarly, these data can help clarify the dietary mechanical landscape of extinct hominins often overlooked by broad C3/C4 isotopic categories.

## Abstract (web summary)

Adam van Casteren et al. report that chimpanzee foods in savannah woodlands are more mechanically demanding than those in tropical forests. This study, based on carbon and nitrogen isotope signature analysis and measurements of the mechanical properties of common food sources, suggests that chimpanzees may adapt their dietary niche to environmental conditions.

## Introduction

Diet is integral to understanding the behaviours and adaptations of extant and extinct primate species alike. Nowhere is this more salient than in the evolution of the hominin tribe and the emergence of modern day humans, as the majority of dietary inferences must be constructed from a patchwork of fossilised craniodental remains. Food mechanics are likely a substantial driver in the adaptation of the dental complex and the constraints that these place on the efficiency of food processing. Understanding how the form of teeth relates to their function therefore requires a synthesis of knowledge over both tooth structure and the mechanical properties of the critical foods that resist being broken down^1^.

In chimpanzees (*Pan troglodytes*), direct behavioural observation and indirect methods such as isotopic and faecal analysis have allowed a rather in-depth knowledge of *what* their diets are composed of^2–10^, and thus allow for some comparison with the putative diets of the earliest hominins^11^. However, in such studies, foods are still largely categorised in very broad terms (e.g., fruits, leaves, bark) that do not faithfully track their mechanical properties^12^. In addition, accessing foods often includes the removal of external tissues with the teeth to access the nutrients within. The mechanical properties of such tissues can vary substantially and can instigate distinct oral feeding practices. Such processing is termed ingestion, which is often facilitated by the anterior dentition and is distinct from mastication, where food is cyclically processed by posterior dentition before being swallowed^13^. The mismatch between the mechanical characteristics of foods and how they are processed orally often makes it difficult to understand the physical conditions that foods exert on teeth and can lead to an oversimplification of this vital interface. Therefore, comparative studies of ingestive behaviours and food mechanical properties in large bodied apes, like chimpanzees, are essential to fully understand relationships between craniodental form and function in fossil hominins.

**Fig. 1:**
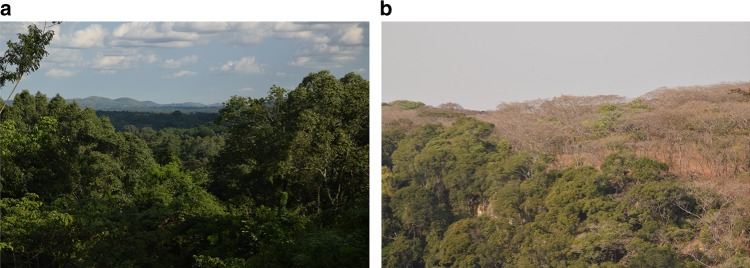
The overt differences in habitat structure where the two distinct chimpanzee communities of this study inhabit. Ngogo (**a**) is a tropical and subtropical moist broadleaf forest where tree species transition between montane and lowland forest. Issa Valley (**b**) is a tropical and subtropical savannahs, grasslands, and shrub lands biome dominated by central Zambezian Miombo woodlands. Photo credit to AvC

Chimpanzees allow for an interesting comparison of feeding in two evolutionarily relevant hominin habitats. The tropical forest is analogous to the original stem hominin habitat^14^, whilst in comparison the savannah woodland mirrors the ecological conditions that drove later hominin adaptation and the emergence of *Homo*^15^ (Fig. 1). Currently our understanding of chimpanzee dentition and its functional aspects are limited by a lack of data on the broader dietary mechanical challenges faced species-wide^16,17^. In fact, data on the mechanical properties effectively hail from one tropical forest^18^, and it is doubtful these values accurately reflect the dietary variance of the species. Unlike forest-dwelling chimpanzees, savannah chimpanzees tend to incorporate and rely upon many non-fruit items^19^. Isotopic studies conducted on chimpanzee populations have established the species firmly in the C_3_ feeding category, meaning that in all habitats chimpanzees primarily feed on tree products that utilise a C_3_ photosynthetic pathway^20–23^. Continued isotopic research has indicated that across chimpanzee habitats, from rainforest to savannah, the values of δ^13^C and δ^15^N vary significantly^21,22^. These patterns are thought to occur because savannah chimpanzees rely more on plant foods produced under drier environments with reduced canopy cover compared to those of their forest counterparts. However, it remains unclear if utilising foods from different environments affects food material properties in different chimpanzee populations and how this is related to isotopic signatures.

Such a relationship could prove invaluable for reconstructing the diets of extinct hominins. Early hominins, with the exception of *Homo*, show increasing craniodental robusticity over time^11,24^. Such morphological change is thought to represent, at least in part, adaptation to more mechanically challenging foods^25–29^. This seemingly correlates well with a broadening of hominin diets over evolutionary time, as demonstrated by the incorporation of a greater percentage of C_4_ resources^11^. However, the instigation of this adaptive morphology predates the incorporation of large amounts of C_4_ resources into the hominin diet^11,24,30–32^. This may indicate that the dietary mechanical pressures that predisposed early hominins to increased craniodental robusticity are in fact to be found in C_3_ as well as C_4_ food resources of the mosaic woodland environment.

To investigate the mechanical variance in chimpanzee diets, we measured the mechanical properties of commonly consumed plant foods of two communities inhabiting rather disparate environments. This dataset was paired with carbon and nitrogen stable isotope data from plants and hair to determine whether isotopic differences were related to mechanical variance. We hypothesised that even accounting for plant baseline, isotopic signatures will be distinct between the two chimpanzee populations and the utilisation of different biomes will promote the oral processing of more mechanically challenging foods by the savannah chimpanzees of Issa, Tanzania, compared to the rainforest population of Ngogo, Uganda.

## Results

### Stable isotope data

**Fig. 2:**
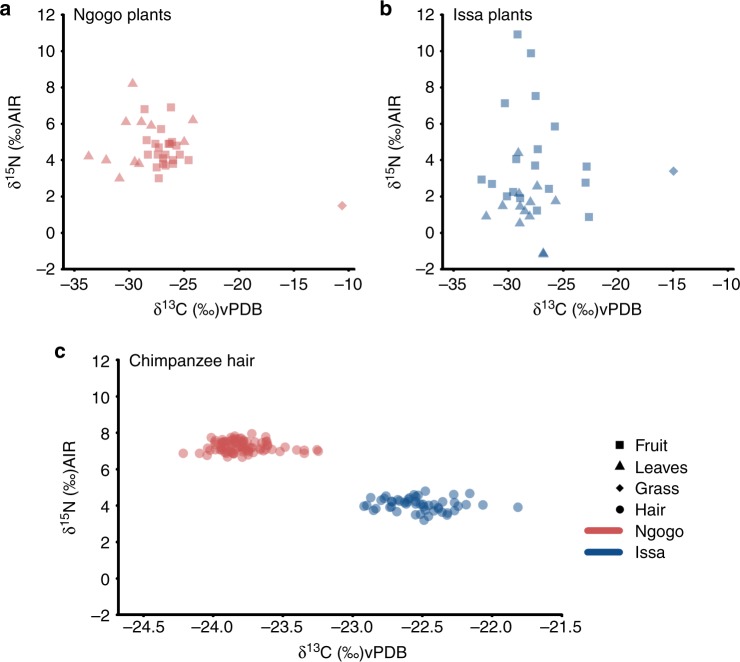
Bivariate plot showing the δ^13^C and δ^15^N values in **a** Ngogo plants categories^33^, **b** Issa plant categories and **c** chimpanzee hair. Analytical errors are smaller than the depicted data points. Despite similar isotopic signals in the plant isotopic signals at both sites results from hair show significant separation in both the δ^13^C values and the δ^15^N values (δ^13^C values: *χ*^2^ = 61.45, df = 1, *p* < 0.0001 and the δ^15^N values *χ*^2^ = 80.67, df = 1, *p* < 0.0001). This indicates that the chimpanzee communities at these two sites utilise foods from distinct habitat types

We found that with a mean of 3.0‰, the δ^15^N plant values at Issa are lower than what is commonly found in chimpanzee habitats. For Ngogo plants, Carlson^33^ reported a mean of 4.5‰ (*n* = 246). A comparison between the δ^15^N values of the two plant datasets controlling for sample type (fruit or leaves) and plant species revealed these differences in δ^15^N are significant between Issa and Ngogo plant foods (*χ*^2^ = 7.36, df = 1, *p* = 0.006) (Fig. 2a, b). However, the same comparison between δ^13^C plant values from Issa and Ngogo^33^ revealed that on the broad scale the sites were indistinguishable in carbon (*χ*^2^ = 0.13, df = 1, *p* = 0.714) (Fig. 2a, b). Samples of the sedge family *Cyperaceae* from Ngogo had a high mean δ^13^C value of −11.6 ‰, whereas the single grass sample we measured from Issa had a more typical C_4_ plant value of −15‰ (Table 1).

**Table 1:** Descriptive statistics for plants and chimpanzee isotope values from Issa and Ngogo

	All plants		Fruit		Leaves		C4 grass		Hair		Δ _plant–hair_	
	δ^15^N	δ^13^C	δ^15^N	δ^13^C	δ^15^N	δ^13^C	δ^15^N	δ^13^C	δ^15^N	δ^13^C	Δ^15^N	Δ^13^C
Issa												
Mean	3	−27.6	4.2	−27.8	1.3	−28.5	3.4	−15	4.1	−22.5	1.0	5.1
Stdev (1*σ*)	2.8	3.3	2.9	2.8	1.5	1.7	—	—	0.4	0.2		
Ngogo												
Mean	4.7	−27.1	4.6	−26.8	5.1	−29.2	1.5	−11	7.2	−23.8	2.6	3.3
Stdev (1*σ*)	1.3	3.6	1	1	1.5	2.8	—	—	0.3	0.2		

We obtained novel δ^13^C and δ^15^N values for a total of 51 hair sections (obtained from 11 individuals) from the Issa chimpanzees and 85 hair sections (obtained from 13 individuals) for Ngogo. Means and standard deviation as well as fractionation factors between mean isotope values of plants and chimpanzee hair isotope values (Δ_plant–hair_) are shown in Table 1.

Both data sets covered the different seasons of the year in an attempt to deliver an annual isotopic spectrum of adult chimpanzees at both sites. Average temporal isotopic variation within individual hair samples is moderate at Ngogo (0.32‰ in δ^13^C, 0.40‰ in δ^15^N) and also at the savannah site of Issa (0.38‰ in δ^13^C, 0.46‰ in δ^15^N); this difference in variation between sites is much smaller than the analytical error and thus not biologically meaningful. This conformity between sites was not expected given the substantial differences in annual rainfall patterns; as one would assume more striking effects of seasonality in the Issa population than in Ngogo. Our model results (see Methods for details) show that the differences between chimpanzees from Ngogo and Issa were highly significant in the δ^13^C values (*χ*^2^ = 61.45, df = 1, *p* < 0.0001) and the δ^15^N values (*χ*^2^ = 80.67, df = 1, *p* < 0.0001), with Issa chimpanzees being less depleted in ^13^C, and much lower in ^15^N (Fig. 2c, Table 1).

### Biomechanical data

At both sites combined, we made 829 (Ngogo *n* = 488 and Issa *n* = 341) measurements of toughness (*R*) and 557 (Ngogo *n* = 321 and Issa *n* = 236) measurements of elastic modulus (*E*) on foods that were orally processed. These measurements included 17 plant species from Ngogo (Table 2) that comprised all species observed above 1% of the feeding time of chimpanzees during 36 h of dry season focal feeding observations. These species feeding times agreed well with long-term observations of the dry season at this site^8^. At Issa, 19 species were tested, including samples from *Ficus*, *Saba*, and *Garcinia* (Table 3) that are considered year-round staple foods^10^. In the dry season at Issa, chimpanzees are thought to rely more on the woodland plant genera, and our sample reflected this with the inclusion of eight mainly woodland species.

**Table 2:** Results from Ngogo displaying averages and standard deviations of *R* and *E* for tissues of different plant species tested

Species	*R* (J m^−2^)	*n*	sd	*E*_i_ (MPa)	sd	*E*_∞_ (MPa)	sd	*n*	*E*_*∞*_ */E*_i_
Exocarp									
* Ficus bracylypis*	206.7	15	59.0	—	—	—	—	—	—
* Ficus capensis*	580.4	5	131.2	—	—	—	—	—	—
* Ficus dawei*	289.8	10	122.5	0.4	0.4	0.3	0.4	7	0.7
* Ficus mercuso*	246.6	35	90.3	1.2	0.6	0.8	0.4	20	0.8
* Ficus pericifolia*	—		—	1.9	1.0	1.2	0.3	4	0.7
* Pseudospondis microcarpa*	611.7	5	117.5	—	—	—	—	—	—
* Pterygota mildbraedii*	1056.6	5	142.6	3.6	0.6	2.7	0.4	5	0.8
* Uvariopsis congenensis*	196.3	8	49.0	0.1	0.1	-	-	6	-
* Zanha golungensis*	875.7	10	281.8	2.1	1.4	1.6	1.2	5	0.7
Mesoderm									
* Aphania senegalensis*	31.4	20	10.8	0.4	0.2	0.3	0.2	15	0.7
* Ficus bracylypis*	164.3	20	88.1	0.6	0.5	0.4	0.4	19	0.6
* Ficus capensis*	712.8	4	59.0	—	—	—	—	—	—
* Ficus dawei*	311.4	20	333.6	0.6	0.9	0.4	0.7	15	0.6
* Ficus mercuso*	120.6	49	69.5	0.5	0.6	0.4	0.5	50	0.7
* Ficus pericifolia*	129.0	20	43.0	0.4	0.1	0.3	0.1	20	0.7
* Morus mesozygia*	664.1	15	291.8	0.6	0.5	0.4	0.3	35	0.7
* Pseudospondis microcarpa*	227.4	5	46.4	0.5	0.1	0.3	0.1	6	0.6
* Pterygota mildbraedii*	—	—	—	0.0	0.0	—	—	5	—
* Zanha golungensis*	—	—	—	0.2	0.1	0.1	0.1	10	0.7
Flowers									
* Antiaris toxicalia*	141.5	8	80.0	0.5	0.6	0.3	0.4	4	0.6
* Pterygota mildbraedii*	296.2	20	131.2	2.6	2.0	1.5	0.5	10	0.7
Pith									
* Afromumum*	780.2	12	137.7	4.4	1.6	1.8	0.5	12	0.4
Leaf laminar									
* Antiaris toxicalia*	359.1	5	45.3	4.5	2.3	4.1	2.3	6	0.9
* Celtis africana*	119.3	23	49.7	—	—	—	—	—	—
* Celtis mildbraedii*	123.2	10	43.8	47.8	22.7	43.0	21.5	9	0.9
* Ficus exasperata*	572.4	15	278.3	27.7	12.1	24.0	11.0	15	0.9
* Ficus varifolia*	284.1	28	178.1	8.6	6.3	7.5	5.9	20	0.9
* Pterygota mildbraedii*	306.1	20	257.1	20.5	9.9	19.9	9.6	23	1.0
Leaf Midrib									
* Celtis africana*	840.6	24	504.8	—	—	—	—	—	—
* Celtis mildbraedii*	648.0	20	165.9	—	—	—	—	—	—
* Ficus exasperata*	4167.2	8	935.0	—	—	—	—	—	—
* Ficus varifolia*	1507.2	24	882.1	—	—	—	—	—	—
* Pterygota mildbraedii*	3715.3	25	2352.9	—	—	—	—	—	—

**Table 3:** Results from Issa displaying averages and standard deviations of *R* and *E* for tissues of different plant species tested

Species	*R* (J m^−2^**)**	*n*	sd	*E*_i_ (MPa)	sd	*E*_∞_ (MPa)	sd	*n*	*E*_∞_ */E*_i_
Exocarp									
* Ficus sp*.	174.8	6	60.7	—	—	—	—	—	—
* Ficus sp. 4*	227.2	5	121.0	—	—	—	—	—	—
* Ficus lutea*	391.0	10	191.2	—	—	—	—	—	—
* Keetia sp*.	384.7	11	182.1	1.2	0.3	—	—	5	—
* Garcinia huillensis*	823.4	13	252.6	2.7	1.5	1.5	0.8	10	0.6
* Grewia rugosifolia*	904.5	13	240.4	—	—	—	—	—	—
* Julbernardia globliflora*	10675.6	20	1802.4	465.7	159.0	—	—	25	—
* Julbernardia unijugata*	25525.6	2	—	203.6	54.3	—	—	5	—
* Parinari curatellifolia*	653.9	20	164.2	8.3	4.9	6.1	3.5	20	0.8
* Pterocarpus tinctorius*	791.8	11	308.4	3.0	1.3	2.0	1.1	10	0.7
* Saba comorensis*	1073.6	6	233.9	1.1	0.6	0.8	0.4	6	0.7
* Strychnos pungens*	6962.8	3	3130.1	31.5	13.1	19.4	12.8	4	0.6
* Strychnos sp*.	10178.6	15	3641.9	22.3	11.6	11.2	8.3	22	0.5
* Uapaca kirkiana*	748.8	11	347.2	6.2	2.2	5.7	2.4	5	0.9
* Ximenia caffra*	481.2	5	59.6	—	—	—	—	—	—
Mesoderm									
* Ficus sp*.	105.9	10	56.7	0.3	0.1	0.2	0.1	10	0.7
* Ficus sp.3*	49.1	6	25.8	0.2	—	—	—	—	—
* Ficus sp.4*	62.1	10	22.2	0.2	0.1	0.1	0.0	10	0.6
* Ficus lutea*	472.7	12	185.5	1.6	1.5	0.7	0.5	6	0.5
* Ficus varifolia*	153.8	17	58.8	0.2	0.3	-	-	15	-
* Garcinia huillensis*	109.3	12	54.5	0.5	0.2	0.2	0.1	10	0.5
* Parinari curatellifolia*	21.5	21	12.7	0.1	0.1	0.1	0.0	20	0.5
Unknown climber	13.1	6	2.9	0.2	0.1	0.1	0.1	5	0.7
* Ximenia caffra*	24.7	4	17.3	0.5	0.7	0.1	0.1	4	0.4
Endosperm									
* Julbernardia globliflora*	920.0	11	210.8	10.6	4.8	9.1	4.3	11	0.8
* Pterocarpus tinctorius*	308.5	9	95.3	4.4	3.5	3.1	2.8	10	0.6
Leaf laminar									
* Syzygium guineense*	180.5	10	96.3	3.8	1.8	3.6	1.8	3	0.9
* Julbernardia globliflora*	184.2	8	79.1	17.7	10.5	17.3	10.5	10	1.0
* Ficus exasperata*	242.0	5	46.4	8.9	3.3	5.7	2.6	5	0.7
* Pterocarpus tinctorius*	94.4	10	34.3	5.2	4.2	4.6	3.8	5	0.9
Leaf Midrib									
* Syzygium guineense*	497.2	10	204.5	—	—	—	—	—	—
* Pterocarpus tinctorius*	639.4	10	419.4	—	—	—	—	—	—
* Ficus exasperata*	807.8	5	328.8	—	—	—	—	—	—
* Julbernardia globliflora*	4338.6	8	4295.6	—	—	—	—	—	—
* Ficus sp.3*	4115.7	6	1336.9	—	—	—	—	—	—

**Fig. 3:**
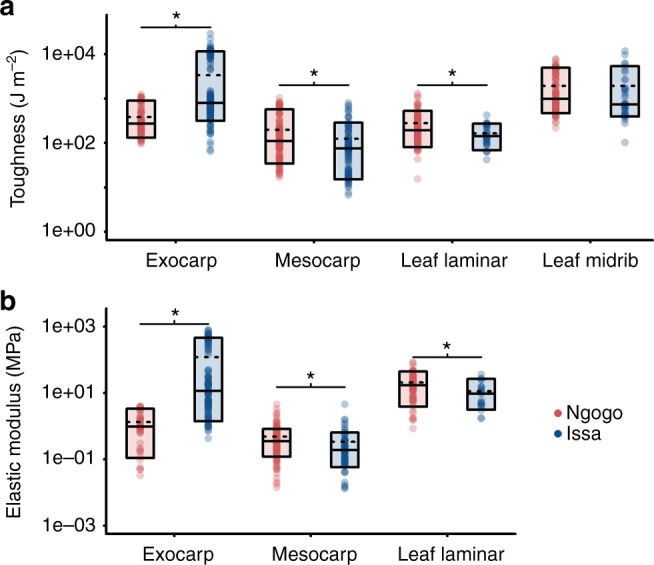
The toughness (**a**) and elastic modulus (**b**) of broad food categories indicates variance between Ngogo and Issa, particularly in the exocarps of fruits. *Y*-axis is a logarithmic scale. Means represented by dashed line and medians represented by solid lines, boxes represent 10th and 90th quartile. Asterisks represent the results of a Mann–Whitney *U* tests between plant tissues categories for both toughness (Exocarp, *W* = 2633.5, *p* < 0.001; Mesocarp, *W* = 9934, *p* < 0.001; Leaf laminar, *W* = 2265, *p* = 0.007; Leaf midrib, *W* = 2267, *p* = 0.1703) and elastic modulus (Exocarp, *W* = 442.5, *p* < 0.001; Mesocarp, *W* = 97705, *p* < 0.001; Leaf laminar, *W* = 1157, *p* = 0.005)

Values for *R* and *E* of orally processed foods overlapped between the two sites. However, there was a noticeable difference, particularly in the range of the values. At Ngogo, toughness ranged from 15 to 7694 J m^−2^, with 0.014–82 MPa for the elastic modulus, but at Issa, both toughness and elastic modulus could be much higher: 6.7–28,869.2 J m^−2^ toughness and 0.013–799 MPa for the elastic modulus. The data were then broken down into food tissue categories (Fig. 3) to help elucidate what may be driving the differences in food mechanics between sites. Values within comparable categories had similar ranges in each location that fell within the values previously published for primate food mechanical properties^18,34,35^. The higher toughness values at Issa were significant for fruit exocarp (Mann–Whitney *U* test, *W* = 2633.5, *p* < 0.001). Lower values recorded for mesocarp at Issa were also significantly so (*W* = 9934, *p* < 0.001). Similarly, values in leaf laminar tissues were significantly lower at Issa (*W* = 2265, *p* = 0.007), yet there was no significant difference for the toughness of leaf midrib (*W* = 2267, *p* = 0.1703). There were also differences in the recorded *E* of the comparable food tissues. The exocarps of fruits from Issa were significantly stiffer than those from Ngogo (*W* = 442.5, *p* < 0.001), whilst the fruit mesocarp from Issa was of a significantly lower stiffness than those of Ngogo (*W* = 97705, *p* < 0.001). A similar relationship was observed for leaf laminar tissue (*W* = 1157, *p* = 0.005). Recorded values of both *R* and *E* demonstrate the most extreme disparity in the exterior casings of fruits that must be breached to obtain nutrient rich mesocarp (see video S1). In Issa exterior tissues such as fruit exocarp demonstrate considerably higher values than are seen in other plant tissues.

**Fig. 4:**
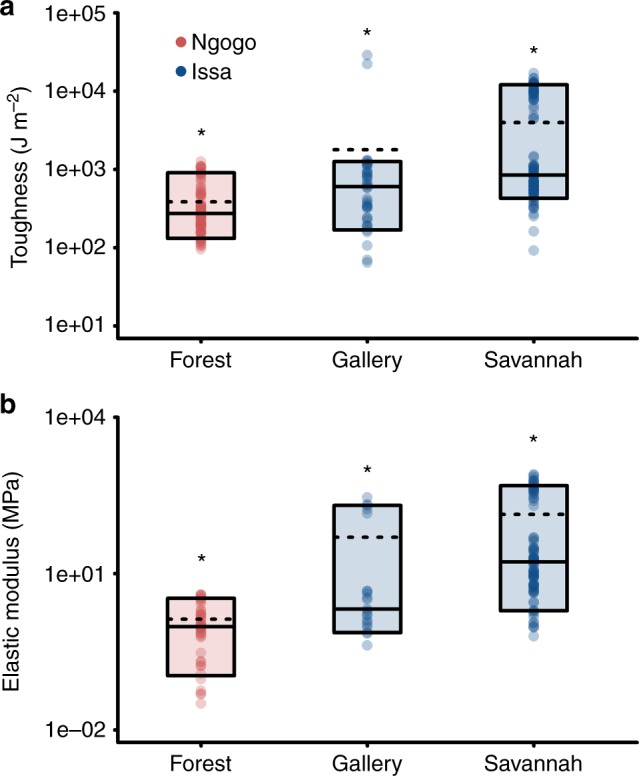
The savannah exocarps of Issa are often tougher (**a**) and stiffer (**b**) than those of the gallery forest patches and the tropical forest. *Y*-axis is a logarithmic scale. Means represented by dashed line and medians represented by solid lines, boxes represent 10th and 90th quartile. Asterisks represent the results of Kruskal Wallis tests for both toughness (*χ*^2^ = 79.3, *p* < 0.001) and elastic modulus (*χ*^2^ = 78.8, *p* < 0.001)

Figure 4 provides a more in depth exploration of the external food casings. The Ngogo study area is mostly covered by moist evergreen and semi-deciduous forest from which all the foods in this study were sampled; therefore, all Ngogo exocarp data were pooled and labelled as forest species. However, the external casings from Issa, a mosaic habitat with multiple biomes, have been broken down into fruits from the gallery forest or fruits from the savannah woodland species. Here it is clear that the largest differences in both *R* and *E* were found in savannah woodland fruits. There was a significant difference between the three categories (forest fruits, gallery forest fruits and savannah woodland fruits *R*, Kruskal Wallis test: *χ*^2^ = 79.3, *p* < 0.001 and *E*, *χ*^2^ = 78.8, *p* < 0.001). A Dunn’s test of multiple comparisons showed that all categories were significantly different from each other in both toughness and stiffness.

## Discussion

Mechanical data from foods consumed by *P. troglodytes schweinfurthii* in Ngogo conformed well to those of Vogel et al.^18^ measured from chimpanzee populations at Kanyawara. All Ngogo data for toughness and stiffness remained at relatively low levels (Fig. 3a, b). Similarities between Kanyawara and Ngogo are not surprising, as both sites are within the Kibale National Park, with foods comprising of comparable plant species. Indeed, there is up to 73% overlap in feeding species between the two sites^3,7^. Ngogo and Kanyawara provide an example of tropical rainforest, and the chimpanzees at both sites have diets comprised largely of fleshy ripe fruits even in times of reduced production^3,8^. This pattern of high fruit consumption characterises chimpanzees inhabiting tropical and subtropical moist broadleaf forests^21^. In such biomes it is likely that lower seasonality and higher fruit availability compared to savannah woodland sites means that the majority of oral processing reduces the mechanical challenges to teeth. At Issa, the mesocarp of fruit, leaf laminar tissue, and leaf midrib also manifested within this rather narrow range (Fig. 3a, b). These are all tissues that are likely masticated by molars and then subsequently swallowed. Previous hypotheses surrounding the molar morphology of *Pan* have suggested that gracile molars with thin enamel are a derived trait adapted for comminuting large amounts of easy to process foods, along with some (possibly seasonal) fracture resistant foods, such as foliage^18^. The loss of thick enamel is likely due to a relaxation of selection pressures that necessitated strong durable crowns adapted to either hard and/or abrasive food tissues. Our data go some way to supporting this hypothesis, demonstrating a lack of variance in the mechanical properties of tissues likely masticated by chimpanzees across our study sites.

Despite the overlap in masticated tissues, data from Issa presents a divergence from this mechanical dietary uniformity. Substantial differences occur in both the toughness (Fig. 4a) and elastic modulus (Fig. 4b) in the external casing of savannah fruits at Issa. Such mechanically challenging tissues will necessitate ingestive processing to access consumable tissues (see for example Supplementary Movie 1). It is noteworthy that the mean values for these tissues, *R* = 1794.0 (sd 5435.2) J m^−2^ and *E* = 50.1 (sd 91.3) MPa, exceed those of Bornean orangutans (*R* = 1152.9 J m^−2^ and *E* of 3.08 MPa, respectively), which are generally considered to consume the most mechanically challenging diet of all the great apes^36^. At Issa, the highest values were generated primarily by the woody valves of *Julbernardia* sp., which are comparable values to other woody legume pods eaten by primates^1,37^ and the resilient outer exocarp of *Strychnos* sp. (Table 3). The exocarp of other savannah species within our data set also demonstrated generally higher toughness and stiffness estimates when compared to foods from rainforest, gallery forest, and values obtained from the literature^1,18,36^ (Fig. 3a, b). We know very little about the mechanical properties of savannah plants, but these are likely to demonstrate a greater variability, as such plant species must have different adaptations to water stress events. All this would indicate that when feeding is more concentrated in the savannah woodland habitats, as is the case at Issa during the dry season^10^, chimpanzees face external plant tissues that are substantially more demanding than those encountered by their conspecifics within Kibale National Park.

Whilst extractive and percussive foraging behaviours are reported in savannah chimpanzee populations^38–41^, the majority of plant foods are likely processed orally. In chimpanzees, oral ingestive processing is done primarily by the anterior teeth^2,38,42^ (an example of such action can be found in Supplementary Movie 1). These feeding behaviours coincide well with morphology, as high forces and unique loading regimes are likely to be necessary in the husking of more mechanically challenging foods, such as those demonstrated at Issa. Unlike basal Miocene apes and later members of the human ancestral clade, chimpanzees have quite derived anterior teeth, most obviously in the size and morphology of the incisors^43^. Chimpanzee incisors are characterised by their large broad spatulate shape^16,42–44^, presenting a more procumbent posture and sharp cutting edge, maintained through a thinning of the lingual enamel, which may also reduce stress on the crown during ingestion^45^. Uniquely amongst hominoids, the lower incisors have converged to the morphology of the uppers^43^. Both upper and lower incisors therefore offer a large optimally angled cutting tool, well adapted for initiating and propagating fracture in foods^46^. Furthermore, each anterior tooth is anchored by a markedly long and large tooth root^47^, making them well equipped to deal with high forces that are likely inflicted on these teeth during the ingestive processing of mechanically challenging foods. It therefore appears that the anterior teeth of chimpanzees form the workhorse of the chimpanzee dentition, and are well adapted to deal with mechanical challenges arising from foods. These teeth are likely utilised to overcome the higher mechanical challenge presented by the external casings of savannah plants within the Issa environment. Such external barriers must be breached in order to gain access to internal nutrient tissues.

Isotopic signatures measured from chimpanzee hair samples show a significant difference between sites in δ^15^N and δ^13^C values (δ^13^C values: *χ*^2^ = 61.45, df = 1, *p* < 0.0001 and the δ^15^N values *χ*^2^ = 80.67, df = 1, *p* < 0.0001). These differences become apparent in the substantial differences in Δ_plant–hair_ isotope values, which show the behavioural difference in chimpanzee habitat utilisation when controlling for isotopic baseline effects in potential plant foods. For δ^15^N, this discrepancy can be explained by the significant differences in the plant isotope baselines between sites (*χ*^2^ = 7.36, df = 1, *p* = 0.006), which are probably driven by the isotope values of non-fruit items such as leaves (see Table 1). This indicates that previous attempts^20,21^ to explain the relatively low δ^15^N values in the Issa chimpanzees in the absence of plant baseline data require revision. Low δ^15^N values in the Issa chimpanzees are best explained by generally depleted plant baseline values in this woodland mosaic habitat, and not necessarily by the chimpanzees’ heavy consumption of nodulating (soil nitrogen fixating) plants. Moreover, an overall depleted δ^15^N signature seems to be more common in savannah chimpanzee sites than previously assumed, as this low δ^15^N pattern has also been observed at several other savannah chimpanzee sites across Africa, including Kayan in Senegal^22^, as well as in several unpublished datasets from West Africa (Oelze personal communication).

In this study, however, we focussed on the site specific signatures in δ^13^C, as they are highly relevant for understanding paleodiets in the fossil record. Measurements of δ^13^C can be obtained from ancient dental enamel, whereas the analysis of δ^15^N is limited to well-preserved organic material containing substantial amounts of nitrogen. The plant δ^13^C values in our study indicate that on a general scale, the isotopic variance between the two habitats is minimal. However, chimpanzee hair isotope values significantly differ in δ^13^C. This evinces to two main outcomes. Firstly, chimpanzees do not always simply resemble the isotopic characteristic of the environment they inhabit, but they have feeding preferences and select microhabitats suitable to meet their dietary demands. Our δ^13^C data suggest that Issa chimpanzees do not feed solely on plant foods (mainly ripe fruits and smaller quantities of leaves) derived from dense gallery forest patches, but rely on ^13^C enriched plants in the open areas of the woodland savannah, which is concurrent with observational and faecal analysis at Issa^4,10^. This is in line with isotopic evidence reported from chimpanzees and their plant foods at the savannah site of Kayan in Senegal^22^ and with what can be assumed from work at other savannah sites like Fongoli, although respective δ^13^C plant data are not yet available^23^. Secondly, δ^13^C values from hair samples differed between sites, but this variance does not resemble the vast differences reported between C_4_ (savannah) and C_3_ (forest) dependent fossil hominin species in East Africa^11,48^, primarily because no known population of chimpanzees has been found to habitually consume C_4_ plant foods^23^. Yet it appears these smaller scale differences may have rather large implications in the acquisition of food and the mechanical challenges encountered in contrasting biomes. Such subtle differences could therefore be of interest to paleoanthropologists reconstructing diets of the past.

A somewhat restrictive diet dominated by C_3_ plants—as found in chimpanzees^11,49^—is often assumed to be somewhat mechanically narrow, i.e., associated with easy to process fruits and forest products. Our data indicate that this is not always the case. Plant tissues consumed by chimpanzees that utilise a C_3_ photosynthetic pathway can demonstrate pronounced mechanical variance and challenges. Broad and easily observable isotopic categorisations based on photosynthetic pathways are critical to our understanding of paleo-environments, but alone these proxies may offer little indication of the finer scale mechanical behaviour of plant foods; it is this which is likely to be driving the adaptations of the craniodental complex of African Plio-Pleistocene fossil hominins.

Although discussion is ongoing concerning the exact paleoenvironment that the australopiths of Pliocene East Africa inhabited, there is some consensus that this niche was either wooded shrubland or wooded grassland, similar to the mosaic savannah woodland environment of extant savannah chimpanzees^15,50^. Fossil findings have also indicated that members of *Pan* have long used these habitat types in sympatry with early *Homo*, a relationship with the human lineage that may have endured since the divergence of *Pan* and hominins^51^. Middle Pliocene australopiths such as *Ardipithicus ramidus* and *Australopithecus anamensis* possess remarkably comparable isotopic signatures with savannah chimpanzees, suggesting they relied on a C_3_ dominated diet^49,52,53^. Whilst perhaps savannah chimpanzees are an imperfect morphological analogy for these early hominins, there are some dental and gnathic similarities (e.g. increased procumbancy and larger incisors) that appear somewhat reduced in later occurring Pliocene hominins (such as *Au. afarensis*) and even more so in Pleistocene hominins (such as *Homo* and *Paranthropus*)^32,54,55^. The coupling of our mechanical and isotopic data suggests that savannah dwelling members of *Pan* that utilise similar habitats and eat mechanically similar foods to our earliest relatives could provide a reasonable extant analogue for exploring early hominin feeding ecology. Further to this, our results indicate that there may have been a shift towards more mechanically challenging foods associated with the hominin transition to exploiting more wooded environments that likely predates the general hominin trend for increased C_4_ consumption.

Our quantitative results of food mechanical properties indicate that many plant tissues masticated by chimpanzees do inhabit a rather narrow dietary range and could be considered rather easy to process. However, this does not comprehensively represent the extent of chimpanzee diets, as harder to process plant tissues can represent substantial contributions to the diets of some populations. We do not advocate that mechanically challenging food items in the chimpanzee diet are only found in savannah environments or that chimpanzees routinely process such foods at all savannah sites. Indeed, different chimpanzee populations have been shown to use seemingly similar environments quite differently with regard to foraging habits^20^. Rather, we show that the possibility exists that in the resource limited savannah woodland environment, chimpanzees choose different foods, some of which are more mechanically challenging than has been considered the dietary norms for this species^36^. Importantly, these tissues are produced by C_3_ plants, indicating that both C_3_ and C_4_ plants can manifest as mechanically challenging plant tissues and both may be responsible for driving dental adaptation. Mechanically challenging tissues, like the external casings of savannah plants, are probably processed to a large extent with the anterior dentition. These teeth are likely to incur larger and more variable forces than the postcanine teeth, as internal tissues that are masticated present only a limited mechanical challenge. Understanding if there is a functional driver behind morphological features of the teeth of chimpanzees and indeed fossil hominins will require a further expansion of the current knowledge of both food mechanical properties and ingestive behaviours on a pan-African scale to reduce our reliance of mechanical property data from singular sites.

## Methods

### The sites

Two sites chosen for this study were the Ngogo Chimpanzee Project and the Greater Mahale Ecosystem Research and Conservation Project (GMERC, formerly Ugalla Primate Project). Both sites were investigated during the dry season, which in both vicinities is associated with a decrease in fruit production and arguably presents a period of greater dietary stress for the chimpanzee communities^4,7^. Chimpanzee hair samples for isotope analysis were collected opportunistically during a 12+ month study period at Ngogo (2012–2013) and Issa (2013–2014) within the framework of the Pan African Programme (http://panafrican.eva.mpg.de/). They represent the annual spectrum of isotope values at each site. At both sites, the samples represent plants from both wet and dry seasons (as defined below).

Ngogo—The Ngogo study area is situated centrally in the Kibale National Park in south-western Uganda^7,56^. The park consists of an area of 795 km^2^, dominated by moist evergreen, with some seasonally deciduous, forest. Tree species are a transition between montane and lowland forest^7,56^ (Fig. 1a). The area receives high rainfall with the yearly average ranging from 1400 to 1600 mm. This is fairly evenly distributed throughout the year, but dry seasons can be defined as two low rainfall levels between June–July and December–February^7,56^. The study area is home to a chimpanzee population of close to 200 individuals that have been continuously observed since 1995. The chimpanzees are well-habituated allowing direct observation of food selection and feeding behaviours^7^.

Issa—The GMERC is located in the Issa valley that lies 100 km east of Lake Tanganyika. The site is a mosaic habitat dominated by savannah woodland (*Brachystegia* and *Julbernardia*) but punctuated by evergreen gallery forests, swamps and grassland (Fig. 1b). Seasonality is high at Issa with two discrete seasons: a wet (October–April) and dry (May–September). The annual rainfall is lower than at Ngogo, averaging 1220 mm per annum with levels dropping to <100 mm in the months of the dry season^10,57,58^. Research on chimpanzees was first conducted in this region in 2001–2003^4^, with a permanent research presence initiated in 2008 by the GMERC that has since been maintained. The Issa community is considered semi-habituated; current research is focused on a 85 km^2^ study area where genetic analysis has identified 67 individuals^57,58^.

### Sample collection for mechanical properties

Ngogo—As the population is well habituated at this site it was possible to make direct observations of what was consumed by individuals. This information was checked against the substantial literature on chimpanzee diet in the Ngogo study area^7,8,59^ to confirm that the items seen eaten were typical for the time of year and habitat. With such guidance, we determined the most important foods to test by conducting day-long follows of chimpanzees, employing the focal techniques used by Vogel et al.^18^. This entailed picking a focal animal from within the group and recording their behaviour continuously for 10 min. After this period elapsed, another individual was then selected and observed. This way one can garner observations across a large group of individuals^18^.

Knowing what is being eaten allowed the selection of foods for measurements of the mechanical properties of individual tissues either ingested or masticated by chimpanzees. Samples were obtained by two main methods. Foods were either dropped by focal animals, this may be because a plant tissue was not consumed, or it was dropped in the process of eating. However to increase the number of samples for testing, food items were also acquired directly from trees accessed using canopy access techniques^60^ that chimpanzees had been observed feeding in.

Issa—The semi habituated state of the population at Issa does not permit the kind of all-day follows of chimpanzees used at Ngogo. Often finding groups of individuals can take some time and the amount of time following is greatly reduced when compared to Ngogo. This means that direct observations of feeding can be reduced to a matter of minutes per day. Therefore, direct observations were used on an opportunistic basis and foods were collected following confirmation that a certain food item was eaten by the chimpanzees. However, due to the low levels of direct observations we also used information from over 4 years of dietary research conducted at Issa which has identified the major food sources from faecal sieving and direct observations alike^10^ this allowed us to target the most commonly consumed dry season foods. In both sites, whenever foods were selected by humans, efforts were taken to match overt cues of readiness of foods for consumption.

### Mechanical properties testing

We measured two main mechanical properties that are particularly pertinent to the breakdown of food: toughness and elastic modulus. We defined toughness as the energy needed to propagate a crack through a material. An estimation of the energy needed to generate a new surface is made and then this is divided by the actual surface area of one side of the crack. The resulting value is termed *R* with the units of joules per metre squared (J m^−2^)^1,61^. This is integral to understanding how foods resist cracks being initiated and propagated by teeth: foods of higher toughness will be more resilient and harder to breakdown during ingestion and mastication. Toughness has been utilised as a dietary proxy in many studies of primate feeding ecology and has helped understand the interface between teeth and foods^1^. The elastic (Young’s) modulus (*E*) of a material is its resistance to reversible deformation, measured as the stress (force per unit area) that produces a strain (a proportional change in dimensions). This can be estimated from the slope of an initial linear region of a stress–strain curve and has units that are usually given in the megapascal (MPa) range for foods consumed by chimpanzees and other primates^1^.

Whenever possible, foods were separated into broad plant anatomical categories, such as exocarp and mesocarp for fruits, with leaves divided into laminar tissue vs. midrib/veins, concordant with Vogel et al.^18,62^. Samples of these tissues were tested individually. To deal with anisotropy, tests were performed in the direction relevant to feeding. This was determined from feeding remains or video evidence. If this was not possible, multiple orientations were tested. All tests in this study were performed on a portable universal testing machine designed for use in the field (Lucas Scientific FLS-1). This machine consists of a hand-cranked movable crosshead and was equipped with a force transducer to measure the resultant forces and a linear variable displacement transducer that measured accurately movements in the crosshead. The equipment is powered by and interfaces with a laptop computer upon which custom built software allows the calculations of the main material properties of foods. There are a multitude of tests available to measure mechanical properties and the tester houses a range of accessories and rigs that can be employed to measure *R* and *E*. Selection of a test depends partly on the size and shape of food items and components and on how chimpanzees process them. Below, we outline the tests that we used during this study.

*Toughness*: Measuring this required the generation of a fracture. We utilised the displacement-controlled action of blades for this purpose, measuring the force needed to propagate a crack through a given area of material. Use of a blade allowed a fracture to be directed through a heterogeneous specimen, such as a leaf for example, such that it accords with the types of fracture seen on samples eaten by chimpanzees. One of the major causes of error in recording toughness via this method is that the interface between blade and material will generate friction and may lead to an overestimate of toughness if not separated out from fracture. However, such friction can be estimated simply by running a second pass of the blade after a fracture has been formed. The blade needs to pass through an identical displacement, with the work recorded, being not that required to produce a new surface, but rather to overcome frictional interactions. This second pass can be subtracted from the originally recorded energy to give a more accurate figure of fracture toughness^61^.

Bulk food items, such as substantial pieces of fruit flesh, had their toughness estimated by employing the wedge test. A sharp wedge (circa 15°) would be driven into a food specimen of known dimensions for a known displacement, thus generating a crack within it. A second pass, as described above, compensates for the influence of friction. The energy actually used in crack formation, obtained by deducting the work done in the second pass from that in the first, was then divided by the area of the newly created surface to obtain an estimate of the toughness^61^. Sometimes the amount of testable material is too small to be wedged. Such tissues are sheet or rod-like structures. When these circumstances arise, a single blade, or two crossing blades as in a pair of scissors, was used to propagate a crack though a material of known dimensions. Again, a second pass is used to compensate for friction between the blade and food or between the two passing blades^61,63^.

*Elastic Modulus*: Measuring the elastic modulus of primate foods has become far easier in recent years with the onset of developments in indentation methods (for more detail, see Talebi et al.^35^ and van Casteren et al.^64^). Blunt indentation uses hemispherical indenters to measure the elastic modulus of a material quickly and with very little sample preparation. All blunt indent tests follow basic load relaxation conditions: a material is loaded slowly at a consistent rate for around 10 s and the resultant “force ramp” is recorded. After 10 s, the displacement is then held constant whilst measuring decay of the load for a further 90 s or until the load becomes constant. A curve is fitted to this relaxation behaviour allowing the calculation of an instantaneous (*E*_i_) and infinite (*E*_∞_) elastic modulus. These terms effectively represent the upper and lower bounds of a material’s elastic resistance and the ratio of the two values (*E*_∞_/*E*_i_) indicates the rate sensitivity of a material. Whilst neither of these values is an ideal representation of what happens in the mouth for this particular study, we consider *E*_i_ to be a more useful measure when considering ingestion and mastication and is used primarily in this investigation^64^.

We used two types of blunt indent test for this study. The first, a bulk indent test, used a large hemispherical probe (of 3.6 mm radius) for measuring the elastic modulus of bulk food items, like fruit flesh. A sample must be cut so that is stable and has a flat surface normal to the probe. Care must be taken that the sample is sufficiently thick (≥2 mm) and that the indent does not exceed 10% of the sample thickness to avoid influence of the substrate on which it rests^64^. The second test is a membrane test that can be used on sheet-like materials like leaves and, in some cases, a peel-like exocarp of a fruit. A test specimen was clamped between two transparent plates that have aligned circular holes, 2 mm in radius, in their centre. A hemispherical probe of 0.25 mm radius is then used to measure the elastic modulus of a specimen—laminar leaf tissue or some external fruit peels by pressing down on a specimen exactly in the centre of the exposed disc of tissues. In this test, the total deformation needed to be less than the total thickness of the specimen being tested to avoid error. After testing, the material was checked for visible damage to ascertain if there was damage due to cellular collapse; such test results were discarded^35^. Both these blunt indentation tests followed the basic load relaxation method described above.

Some foods cannot be indented because their shape and size does not allow for this, e.g., specimens in the form of rods. In these cases, we resorted to more traditional compression tests where possible. Cylinders of material of known dimension were compressed and the elastic modulus calculated as the slope of the initial region of the stress strain graph^65^. For woody material, or that arranged in a rod-like manner, 4-point bending tests were used to calculate the elastic modulus. This is where a beam of known dimensions is bent and the elastic modulus estimated from the elastic phase of this bending behaviour^65^.

### Stable isotope sampling and analysis

For this study we analysed 11 hair samples from the chimpanzees at Issa, and 13 hair samples from the Ngogo chimpanzees in Kibale. Chimpanzee hair samples exported from Uganda and Tanzania were done so following the regulations set out in the Convention on International Trade in Endangered Species of Wild Fauna and Flora (CITES). CITES Permit No. UG003042 (Uganda) and CITES Permit No. 28753 (Tanzania). Samples consist of at least 10–15 hairs each and were obtained non-invasively from fresh or recent nests (nest decay stage 1 or 2, see Kouakou et al.^66^) (Supplementary Table 1), which were associated to four distinct nests groups at Issa and to five nest groups in Ngogo. By focussing on nest groups we tried to ensure the sampling of different members of a chimpanzee party with the aim to minimise potential errors easily introduced by pseudoreplication^67^. Hair samples were prepared following the procedure outlined in detail by Oelze^68^, with an emphasis on removing potential infant hairs and lipid contaminants from the material used for isotope analysis. All hair used contained root bulbs in the telogen stage and was cut sequentially in 5 or 10 mm long sections as weight for analysis allowed (<3.5 mg). Each hair yielded multiple isotope measurements with hair section isotope values reflecting the previous 2 weeks (5 mm) or one month (10 mm) of diet if human hair growth rates are used as a proxy. As a result, each complete hair sample reaches several months back into time and covers on average six previous months of chimpanzee dietary behaviour^68^.

Plant carbon isotope data from Ngogo were available due to the extensive work of Bryce Carlson and could be extracted from the literature^33^. Although several peer-reviewed publications contain the carbon data from his work, we decided to refer to his PhD dissertation, as it contains both δ^13^C and δ^15^N data on Ngogo plants, reporting means for samples for which multiple samples had been collected. Ngogo plant samples were collected in the different seasons of 2009 and 2010 and represent the top 40 plant foods known to be preferred by the Ngogo chimpanzees^33,69^. To ease the comparison with the Issa plant data, we considered only the data obtained from fruits and leaves (*n* = 184, reported mean isotope values *n* = 34, see Supplementary Table 2), including fruits, seeds, pulp and grasses but excluding roots, bark, flowers, and piths. These plant samples were selected based on the chimpanzees’ feeding preferences and thus encompass the different levels of the canopy as exploited by the Ngogo chimpanzees, including ground and high canopy foods^69,70^. In Issa we collected a small selection of representative plant samples (*n* = 32) for stable isotope analysis in the wet and dry seasons of 2015 and 2016. We focussed on plant foods assumed to be essential for the Issa chimpanzees based on the literature^10^, feeding signs, and the presence of the tree species in the GMERC’s phenology inventory. Thus food plant samples were predominantly obtained from miombo woodland and gallery forest habitat types and much less so from open savannah areas. All Issa plant materials are represented by bulk fruits (exocarp, mesocarp, seeds) and leaves, but also by one sample of grass from the open savannah (Supplementary Table 3). As in Ngogo, plant sampling followed evidence of chimpanzees’ feeding selection and thus encompasses samples from the different layers of the canopy. Ripe fruit and leaves were predominantly collected after being dropped to the ground by various animals feeding in the canopy, whereas some mature leaves and terrestrial herbs such as *Aframomum* sp. and the unidentified grass were collected from the subcanopy level. Both datasets are slightly over representative of fruit over leaves, which we consider to resemble chimpanzee feeding preferences. Plant samples exported from Tanzania were done so with the permission of the Tanzanian Chamber of Commerce, Industry and Agriculture (Permit No. A025760) and adhered to Phytosanitary conditions for export (Phytosantary certificate No. 215903). All plant materials were thoroughly dried, homogenised to a fine powder in a pebble mill, and ~2 mg were weighed into tin capsules for isotopic measurement.

All stable isotope measurements were performed in a Flash 2000 – HAT elemental analyser (Thermo Fisher Scientific, Waltham, USA) coupled via ConFLo IV (Thermo Fisher Scientific, Waltham, USA) with a MAT 253 mass spectrometer (Thermo Fisher Scientific, Waltham, USA) at the commercial stable isotope laboratory IsoDetect in Leipzig, Germany. The stable isotope ratios of carbon (δ^13^C) and nitrogen (δ^15^N) are expressed as the ratio of ^13^C/^12^C and ^15^N/^14^N ratios, respectively, using the delta (δ) notation in parts per thousand or permil (‰) relative to the international standard materials Vienna PeeDee Belemite (vPDB) and atmospheric N_2_. The analytical error calculated from repetitive measurements of international (USGS25, USGS40, and USGS41 for N; IAEA-CH6, IAEA-CH7 and IAEA-CH3 for C) and lab-internal standards (caffeine, methionine) included in each run is less than 0.2 ‰ (2σ) for δ^13^C and δ^15^N. To assure analytical quality we excluded all hair isotope data with atomic C:N ratios outside the acceptable 2.6–3.8 range^71^.

For statistical analysis we used R (version 3.4.1, R Development Core Team 2017. We tested the response variables δ^13^C and δ^15^N in plant samples by running two separate mixed models with Gaussian error structure containing the fixed effect of ‘site’, and the control predictor ‘plant sample’, as well as the random effect of ‘plant species’, accounting for multiple measurements per taxon in the datasets used. We excluded the C_4_ grass samples from both plant datasets in our analysis due to low sample size for this control variable. We calculated *p*-values for both models by comparing a full model against a null model excluding the fixed effect of ‘site’ with the function ANOVA. To compare the δ^13^C and δ^15^N values in chimpanzee hair between sites, we also tested each isotope value as a response in a linear model with Gaussian error structure. In both models we included the main effect of ‘site’ and the random effect of ‘hair sample’ to account for the fact that we conducted several measurements per hair sample and thus per individual. We obtained model results by running an ANOVA with the full model and a null model excluding the main effect. For all the four above models, various diagnostic plots of the residuals against fitted values confirmed normal distribution of residuals in the models. We tested variance inflation factors and found no issues with collinearity. Model stability was tested by running each model again by excluding single observations one at a time and comparing the respective model results. Stability tests showed no sign of influential cases.

### Data availability

The data that support the findings of this study are available from the corresponding author upon reasonable request.

## Electronic supplementary material

Supplementary Material

Description of Additional Supplementary Information

Supplementary Movie 1
