# Supplementary material for: Food mechanical properties and isotopic signatures in forest versus savannah dwelling eastern chimpanzees
Source: Commun Biol. 2018 Aug 10;1:109. doi: 10.1038/s42003-018-0115-6 (PMC6123729; doi:10.1038/s42003-018-0115-6)
Supplement: Supplementary file 1 — Supplementary Material [file 42003_2018_115_MOESM1_ESM.pdf]

Supplementary Table 1: Chimpanzee hair $\delta^{15}N$ and $\delta^{13}C$ data from both sites

site	sample	genus	species	sample_ID	section*	C:N	$\delta^{15}N$	$\delta^{13}C$
Ngogo	hair	*Pan*	*troglodytes schweinfurthi*	Ngo_023_1_7	b	3.2	7.7	-23.8
Ngogo	hair	*Pan*	*troglodytes schweinfurthi*	Ngo_023_1_7	c	3.2	7.5	-23.9
Ngogo	hair	*Pan*	*troglodytes schweinfurthi*	Ngo_023_1_7	d	3.2	7.7	-24.0
Ngogo	hair	*Pan*	*troglodytes schweinfurthi*	Ngo_023_1_7	e	3.2	7.7	-23.8
Ngogo	hair	*Pan*	*troglodytes schweinfurthi*	Ngo_023_1_7	f	3.2	7.9	-23.7
Ngogo	hair	*Pan*	*troglodytes schweinfurthi*	Ngo_023_1_7	g	3.2	7.8	-23.6
Ngogo	hair	*Pan*	*troglodytes schweinfurthi*	Ngo_253_1_2	b	3.1	7.0	-23.5
Ngogo	hair	*Pan*	*troglodytes schweinfurthi*	Ngo_253_1_3	c	3.2	7.2	-23.5
Ngogo	hair	*Pan*	*troglodytes schweinfurthi*	Ngo_253_1_4	d	3.2	7.0	-23.4
Ngogo	hair	*Pan*	*troglodytes schweinfurthi*	Ngo_253_1_2	e	3.1	7.2	-23.7
Ngogo	hair	*Pan*	*troglodytes schweinfurthi*	Ngo_253_1_3	b	3.0	6.7	-23.8
Ngogo	hair	*Pan*	*troglodytes schweinfurthi*	Ngo_253_1_3	c	2.9	6.8	-23.9
Ngogo	hair	*Pan*	*troglodytes schweinfurthi*	Ngo_253_1_3	d	2.9	7.0	-23.8
Ngogo	hair	*Pan*	*troglodytes schweinfurthi*	Ngo_253_1_3	e	2.9	7.0	-23.8
Ngogo	hair	*Pan*	*troglodytes schweinfurthi*	Ngo_253_1_3	f	3.0	7.0	-23.9
Ngogo	hair	*Pan*	*troglodytes schweinfurthi*	Ngo_253_1_3	g	3.0	6.9	-24.1
Ngogo	hair	*Pan*	*troglodytes schweinfurthi*	Ngo_253_1_4	b	3.1	7.0	-23.8
Ngogo	hair	*Pan*	*troglodytes schweinfurthi*	Ngo_253_1_4	c	3.1	6.9	-23.9
Ngogo	hair	*Pan*	*troglodytes schweinfurthi*	Ngo_253_1_4	d	3.0	7.1	-23.7
Ngogo	hair	*Pan*	*troglodytes schweinfurthi*	Ngo_253_1_4	e	3.1	7.0	-23.6
Ngogo	hair	*Pan*	*troglodytes schweinfurthi*	Ngo_253_1_4	f	3.1	7.2	-23.9
Ngogo	hair	*Pan*	*troglodytes schweinfurthi*	Ngo_253_1_4	g	2.9	6.9	-23.9
Ngogo	hair	*Pan*	*troglodytes schweinfurthi*	Ngo_253_1_4	h	2.9	7.1	-23.8
Ngogo	hair	*Pan*	*troglodytes schweinfurthi*	Ngo_253_1_5	b	2.9	7.0	-24.0
Ngogo	hair	*Pan*	*troglodytes schweinfurthi*	Ngo_253_1_5	c	2.9	6.9	-23.9
Ngogo	hair	*Pan*	*troglodytes schweinfurthi*	Ngo_253_1_5	d	2.9	7.0	-23.9
Ngogo	hair	*Pan*	*troglodytes schweinfurthi*	Ngo_253_1_5	e	2.9	7.2	-23.9
Ngogo	hair	*Pan*	*troglodytes schweinfurthi*	Ngo_253_1_5	f	2.8	6.9	-24.2
Ngogo	hair	*Pan*	*troglodytes schweinfurthi*	Ngo_253_1_6	b	2.8	7.0	-23.2
Ngogo	hair	*Pan*	*troglodytes schweinfurthi*	Ngo_253_1_6	c	2.9	6.9	-23.3
Ngogo	hair	*Pan*	*troglodytes schweinfurthi*	Ngo_253_1_6	d	2.9	7.1	-23.3
Ngogo	hair	*Pan*	*troglodytes schweinfurthi*	Ngo_253_1_6	e	2.9	7.1	-23.6
Ngogo	hair	*Pan*	*troglodytes schweinfurthi*	Ngo_253_1_6	f	2.9	7.0	-23.3
Ngogo	hair	*Pan*	*troglodytes schweinfurthi*	Ngo_253_1_6	g	2.9	7.3	-23.6
Ngogo	hair	*Pan*	*troglodytes schweinfurthi*	Ngo_253_1_6	h	2.8	7.1	-23.8
Ngogo	hair	*Pan*	*troglodytes schweinfurthi*	Ngo_481_1_1	b	2.9	7.2	-23.8
Ngogo	hair	*Pan*	*troglodytes schweinfurthi*	Ngo_481_1_1	c	2.8	7.2	-23.9
Ngogo	hair	*Pan*	*troglodytes schweinfurthi*	Ngo_481_1_1	d	2.8	7.1	-23.9
Ngogo	hair	*Pan*	*troglodytes schweinfurthi*	Ngo_481_1_1	e	2.8	7.1	-23.6
Ngogo	hair	*Pan*	*troglodytes schweinfurthi*	Ngo_481_1_1	f	2.8	7.2	-23.7
Ngogo	hair	*Pan*	*troglodytes schweinfurthi*	Ngo_481_1_1	g	2.8	7.2	-23.8
Ngogo	hair	*Pan*	*troglodytes schweinfurthi*	Ngo_481_1_1	h	2.7	6.9	-23.9
Ngogo	hair	*Pan*	*troglodytes schweinfurthi*	Ngo_023_1_1	b	3.5	7.6	-23.9
Ngogo	hair	*Pan*	*troglodytes schweinfurthi*	Ngo_023_1_1	c	3.5	7.4	-23.9
Ngogo	hair	*Pan*	*troglodytes schweinfurthi*	Ngo_023_1_1	d	3.5	7.8	-23.8
Ngogo	hair	*Pan*	*troglodytes schweinfurthi*	Ngo_023_1_1	e	3.5	7.6	-23.8
Ngogo	hair	*Pan*	*troglodytes schweinfurthi*	Ngo_023_1_1	f	3.5	7.5	-23.8

Ngogo	hair	*Pan*	*troglodytes schweinfurthi*	Ngo_023_1_1	g	3.5	7.5	-23.6
Ngogo	hair	*Pan*	*troglodytes schweinfurthi*	Ngo_023_1_1	h	3.5	7.1	-23.7
Ngogo	hair	*Pan*	*troglodytes schweinfurthi*	Ngo_023_1_1	i	3.5	7.5	-23.8
Ngogo	hair	*Pan*	*troglodytes schweinfurthi*	Ngo_023_1_1	j	3.5	7.2	-23.8
Ngogo	hair	*Pan*	*troglodytes schweinfurthi*	Ngo_023_1_1	k	3.6	7.3	-23.9
Ngogo	hair	*Pan*	*troglodytes schweinfurthi*	Ngo_023_1_1	l	3.5	7.3	-23.9
Ngogo	hair	*Pan*	*troglodytes schweinfurthi*	Ngo_023_1_3	b	3.4	7.6	-23.8
Ngogo	hair	*Pan*	*troglodytes schweinfurthi*	Ngo_023_1_3	c	3.4	7.6	-23.9
Ngogo	hair	*Pan*	*troglodytes schweinfurthi*	Ngo_023_1_3	d	3.4	7.5	-23.8
Ngogo	hair	*Pan*	*troglodytes schweinfurthi*	Ngo_023_1_3	e	3.4	7.5	-23.7
Ngogo	hair	*Pan*	*troglodytes schweinfurthi*	Ngo_023_1_3	f	3.4	7.5	-23.6
Ngogo	hair	*Pan*	*troglodytes schweinfurthi*	Ngo_023_1_3	g	3.4	7.3	-23.6
Ngogo	hair	*Pan*	*troglodytes schweinfurthi*	Ngo_023_1_3	h	3.4	7.5	-23.6
Ngogo	hair	*Pan*	*troglodytes schweinfurthi*	Ngo_023_1_3	i	3.4	7.2	-23.7
Ngogo	hair	*Pan*	*troglodytes schweinfurthi*	Ngo_023_1_4	b	3.4	7.4	-23.9
Ngogo	hair	*Pan*	*troglodytes schweinfurthi*	Ngo_023_1_4	c	3.4	7.3	-24.0
Ngogo	hair	*Pan*	*troglodytes schweinfurthi*	Ngo_023_1_4	d	3.4	7.5	-23.9
Ngogo	hair	*Pan*	*troglodytes schweinfurthi*	Ngo_023_1_4	e	3.4	7.2	-23.8
Ngogo	hair	*Pan*	*troglodytes schweinfurthi*	Ngo_023_1_4	f	3.4	6.9	-23.9
Ngogo	hair	*Pan*	*troglodytes schweinfurthi*	Ngo_023_1_5	b	3.4	7.1	-24.0
Ngogo	hair	*Pan*	*troglodytes schweinfurthi*	Ngo_023_1_5	c	3.4	7.1	-24.0
Ngogo	hair	*Pan*	*troglodytes schweinfurthi*	Ngo_023_1_5	d	3.4	7.1	-24.0
Ngogo	hair	*Pan*	*troglodytes schweinfurthi*	Ngo_023_1_5	e	3.4	7.0	-23.9
Ngogo	hair	*Pan*	*troglodytes schweinfurthi*	Ngo_023_1_5	f	3.4	6.8	-23.7
Ngogo	hair	*Pan*	*troglodytes schweinfurthi*	Ngo_481_1_1	i	3.7	6.9	-23.7
Ngogo	hair	*Pan*	*troglodytes schweinfurthi*	Ngo_481_1_1	j	3.7	6.9	-23.7
Ngogo	hair	*Pan*	*troglodytes schweinfurthi*	Ngo_481_1_1	k	3.7	6.8	-24.0
Ngogo	hair	*Pan*	*troglodytes schweinfurthi*	Ngo_481_1_1	l	3.6	6.7	-23.9
Ngogo	hair	*Pan*	*troglodytes schweinfurthi*	Ngo_481_1_2	b	3.6	7.7	-23.9
Ngogo	hair	*Pan*	*troglodytes schweinfurthi*	Ngo_481_1_2	c	3.6	7.5	-23.8
Ngogo	hair	*Pan*	*troglodytes schweinfurthi*	Ngo_481_1_2	d	3.6	7.6	-23.9
Ngogo	hair	*Pan*	*troglodytes schweinfurthi*	Ngo_481_1_2	e	3.6	7.7	-23.8
Ngogo	hair	*Pan*	*troglodytes schweinfurthi*	Ngo_481_1_2	f	3.5	7.4	-23.8
Ngogo	hair	*Pan*	*troglodytes schweinfurthi*	Ngo_481_1_2	g	3.5	7.5	-23.9
Ngogo	hair	*Pan*	*troglodytes schweinfurthi*	Ngo_481_1_2	h	3.5	7.5	-24.0
Ngogo	hair	*Pan*	*troglodytes schweinfurthi*	Ngo_481_1_2	i	3.5	7.6	-24.0
Ngogo	hair	*Pan*	*troglodytes schweinfurthi*	Ngo_481_1_2	j	3.5	7.4	-23.8
Ngogo	hair	*Pan*	*troglodytes schweinfurthi*	Ngo_481_1_2	k	3.5	7.5	-23.9
Issa	hair	*Pan*	*troglodytes schweinfurthii*	UGA_237	b	3.7	3.8	-22.8
Issa	hair	*Pan*	*troglodytes schweinfurthii*	UGA_237	c	3.7	3.7	-22.8
Issa	hair	*Pan*	*troglodytes schweinfurthii*	UGA_237	d	3.7	4.0	-22.9
Issa	hair	*Pan*	*troglodytes schweinfurthii*	UGA_237	e	3.7	4.0	-22.9
Issa	hair	*Pan*	*troglodytes schweinfurthii*	UGA_237	f	3.7	3.9	-22.7
Issa	hair	*Pan*	*troglodytes schweinfurthii*	UGA_237	g	3.7	3.7	-22.7
Issa	hair	*Pan*	*troglodytes schweinfurthii*	UGA_238_A	b	3.5	3.8	-22.5
Issa	hair	*Pan*	*troglodytes schweinfurthii*	UGA_238_A	c	3.6	4.2	-22.5
Issa	hair	*Pan*	*troglodytes schweinfurthii*	UGA_238_A	d	3.6	4.1	-22.5
Issa	hair	*Pan*	*troglodytes schweinfurthii*	UGA_238_A	e	3.6	4.1	-22.5
Issa	hair	*Pan*	*troglodytes schweinfurthii*	UGA_238_A	f	3.6	4.0	-22.5
Issa	hair	*Pan*	*troglodytes schweinfurthii*	UGA_238_A	g	3.6	3.9	-22.4

Issa	hair	*Pan*	*troglodytes schweinfurthii*	UGA_238_A	h	3.6	3.7	-22.3
Issa	hair	*Pan*	*troglodytes schweinfurthii*	UGA_238_A	i	3.6	3.5	-22.3
Issa	hair	*Pan*	*troglodytes schweinfurthii*	UGA_238_A	j	3.6	3.2	-22.5
Issa	hair	*Pan*	*troglodytes schweinfurthii*	UGA_238_B	b	3.6	3.7	-22.4
Issa	hair	*Pan*	*troglodytes schweinfurthii*	UGA_238_B	c	3.6	3.5	-22.5
Issa	hair	*Pan*	*troglodytes schweinfurthii*	UGA_238_B	d	3.6	3.4	-22.5
Issa	hair	*Pan*	*troglodytes schweinfurthii*	UGA_238_B	e	3.6	3.5	-22.5
Issa	hair	*Pan*	*troglodytes schweinfurthii*	UGA_238_B	f	3.6	3.6	-22.3
Issa	hair	*Pan*	*troglodytes schweinfurthii*	UGA_238_B	g	3.6	3.6	-22.4
Issa	hair	*Pan*	*troglodytes schweinfurthii*	UGA_238_B	h	3.6	3.9	-22.5
Issa	hair	*Pan*	*troglodytes schweinfurthii*	UGA_238_B	i	3.6	4.2	-22.7
Issa	hair	*Pan*	*troglodytes schweinfurthii*	UGA_255	b	3.6	4.4	-22.6
Issa	hair	*Pan*	*troglodytes schweinfurthii*	UGA_255	c	3.6	4.6	-22.6
Issa	hair	*Pan*	*troglodytes schweinfurthii*	UGA_255	d	3.6	4.3	-22.7
Issa	hair	*Pan*	*troglodytes schweinfurthii*	UGA_255	e	3.6	4.2	-22.3
Issa	hair	*Pan*	*troglodytes schweinfurthii*	UGA_255	f	3.6	4.1	-22.5
Issa	hair	*Pan*	*troglodytes schweinfurthii*	UGA_256	b	3.7	4.0	-22.1
Issa	hair	*Pan*	*troglodytes schweinfurthii*	UGA_256	c	3.6	3.9	-21.8
Issa	hair	*Pan*	*troglodytes schweinfurthii*	UGA_257	b	3.6	4.2	-22.7
Issa	hair	*Pan*	*troglodytes schweinfurthii*	UGA_257	c	3.6	4.1	-22.6
Issa	hair	*Pan*	*troglodytes schweinfurthii*	UGA_257	d+e	3.6	3.8	-22.4
Issa	hair	*Pan*	*troglodytes schweinfurthii*	UGA_258	b+c	3.6	4.2	-22.6
Issa	hair	*Pan*	*troglodytes schweinfurthii*	UGA_258	d+e	3.6	3.9	-22.7
Issa	hair	*Pan*	*troglodytes schweinfurthii*	UGA_258	f	3.6	4.0	-22.4
Issa	hair	*Pan*	*troglodytes schweinfurthii*	UGA_258	g	3.6	4.2	-22.4
Issa	hair	*Pan*	*troglodytes schweinfurthii*	UGA_278	b	3.6	4.4	-22.9
Issa	hair	*Pan*	*troglodytes schweinfurthii*	UGA_278	c	3.6	4.4	-22.6
Issa	hair	*Pan*	*troglodytes schweinfurthii*	UGA_278	d	3.7	4.6	-22.5
Issa	hair	*Pan*	*troglodytes schweinfurthii*	UGA_279	b	3.7	4.5	-22.5
Issa	hair	*Pan*	*troglodytes schweinfurthii*	UGA_279	c	3.6	4.5	-22.8
Issa	hair	*Pan*	*troglodytes schweinfurthii*	UGA_279	d	3.7	4.2	-22.7
Issa	hair	*Pan*	*troglodytes schweinfurthii*	UGA_279	e	3.6	4.1	-22.2
Issa	hair	*Pan*	*troglodytes schweinfurthii*	UGA_280	b	3.6	4.4	-22.8
Issa	hair	*Pan*	*troglodytes schweinfurthii*	UGA_280	c	3.6	4.3	-22.8
Issa	hair	*Pan*	*troglodytes schweinfurthii*	UGA_280	d	3.6	4.2	-22.6
Issa	hair	*Pan*	*troglodytes schweinfurthii*	UGA_280	e	3.6	4.0	-22.2
Issa	hair	*Pan*	*troglodytes schweinfurthii*	UGA_281	b+c	3.6	4.8	-22.5
Issa	hair	*Pan*	*troglodytes schweinfurthii*	UGA_281	d+e	3.5	4.6	-22.3
Issa	hair	*Pan*	*troglodytes schweinfurthii*	UGA_281	f+g	3.6	4.7	-22.2

* note that the first hair sections with the root (a) is not used for IRMS to enable genetic analysis

Supplementary Table 2: Plant $\delta^{15}N$ and $\delta^{13}C$ data for the rainforest site of Ngogo in Uganda.

**site**	**sample**	**genus**	**species**	**n**	**$\delta^{15}N$***	**$\delta^{13}C$***
Ngogo	fruit	*Aningeria*	*altissima*	5	4.8	-25.7
Ngogo	fruit	*Celtis*	*durandii*	5	6.9	-26.2
Ngogo	fruit	*Chrysophyllum*	*albidum*	9	4.7	-27.3
Ngogo	fruit	*Cordia*	*millenii*	3	4.9	-26.4
Ngogo	fruit	*Ficus*	*brachylepis*	5	4.3	-28.3
Ngogo	fruit	*Ficus*	*dawei*	3	4.0	-26.0

Ngogo	fruit	*Ficus*	*exasperata*	7	4.9	-26.3
Ngogo	fruit	*Ficus*	*mucuso*	9	4.1	-26.9
Ngogo	fruit	*Ficus*	*natalensis*	6	3.8	-26.9
Ngogo	fruit	*Mimusops*	*bagshawei*	6	3.7	-26.7
Ngogo	fruit	*Monodora*	*myristica*	3	5.7	-27.1
Ngogo	fruit	*Monodora*	*myristica*	3	6.8	-28.6
Ngogo	fruit	*Monodora*	*myristica*	5	5.1	-28.4
Ngogo	fruit	*Morus*	*lactea*	5	4.3	-25.4
Ngogo	fruit	*Neoboutonia*	*macrocalyx*	5	5.0	-26.1
Ngogo	fruit	*Pseudospondias*	*microcarpa*	4	3.0	-27.3
Ngogo	fruit	*Pterygota*	*mildbraedii*	6	3.6	-27.5
Ngogo	fruit	*Treculia*	*africana*	4	3.8	-26.0
Ngogo	fruit	*Treculia*	*africana*	2	4.3	-26.7
Ngogo	fruit	*Uvariopsis*	*congensis*	5	4.3	-27.4
Ngogo	fruit	*Warburgia*	*ugandensis*	6	4.0	-24.6
Ngogo	fruit	*Neoboutonia*	*macrocalyx*	5	4.9	-27.6
Ngogo	leaves	*Afromomum*	*sp.*	8	3.9	-29.5
Ngogo	leaves	*Celtis*	*africana*	9	3.8	-29.1
Ngogo	leaves	*Celtis*	*mildbraedii*	3	6.1	-30.3
Ngogo	leaves	*Celtis*	*mildbraedii*	1	8.2	-29.7
Ngogo	leaves	*Chaetacme*	*aristata*	5	6.1	-28.9
Ngogo	leaves	*Ficus*	*exasperata*	12	5.9	-28.0
Ngogo	leaves	*Monodora*	*myristica*	6	4.0	-32.1
Ngogo	leaves	*Morus*	*lactea*	4	5.0	-25.0
Ngogo	leaves	*Morus*	*lactea*	9	4.2	-33.7
Ngogo	leaves	*Morus*	*lactea*	2	6.2	-24.2
Ngogo	leaves	*Pterygota*	*mildbraedii*	9	3.0	-30.9
Ngogo	grass	*Cyperus*	*papyrus*	5	1.5	-10.6

* all data from Ngogo plants after Bryce Carlson PhD disseration 2011,
values are averages in case of multiple samples per measurement

Supplementary Table 3: Plant $\delta^{15}N$ and $\delta^{13}C$ data for the dry habitat site of Issa in Tanzania.

site	sample*	*genus*	*species*	$\delta^{15}N$	$\delta^{13}C$	%N	%C
Issa	fruit	*Canthium*	*sp.*	1.2	-27.4	0.7	49.9
Issa	fruit	*Hexalobus*	*monopetalis*	4.1	-29.3	1.4	49.2
Issa	fruit	*Julbernardia*	*unijugata*	2.4	-26.3	1.1	46.3
Issa	fruit	*Saba*	*comoroensis*	1.9	-28.9	1.1	45.4
Issa	fruit	*Carpolobia*	*goetzei*	2.9	-32.4	0.9	44.2
Issa	fruit	*Vitex*	*sp.*	10.9	-29.2	0.3	44.9
Issa	fruit	*Vitex*	*sp.*	7.1	-30.3	0.3	44.5
Issa	fruit	*Uvaria*	*angolensis*	2.7	-31.5	0.7	50.5
Issa	fruit	*Cordia*	*sp.*	4.6	-27.3	0.6	43.7
Issa	fruit	*Aframomum*	*sp.*	2.3	-29.6	0.7	41.2
Issa	fruit	*Parinari*	*curatellifolia capensis*	7.5	-27.5	0.3	46.5
Issa	fruit	*Parinari*	*curatellifolia capensis*	9.9	-27.9	0.3	46.6
Issa	fruit	*Strychnos*	*innocua*	2.0	-30.1	1.0	42.3
Issa	fruit	*Ficus*	*thonningi*	3.6	-22.9	0.6	47.7
Issa	fruit	*Landolphia*	*owariensis*	3.7	-27.6	1.6	48.2

Issa	fruit	*Garcinia*	*huillensis*	0.9	-22.7	0.9	40.9
Issa	fruit	*Garcinia*	*huillensis*	2.8	-23.0	0.9	41.4
Issa	fruit	*Ficus*	*thonningi*	5.9	-25.8	1.0	48.1
Issa	leaves	*Ficus*	*exasperata*	4.4	-29.1	3.2	42.8
Issa	leaves	*Vitex*	*mombassae*	0.9	-32.0	0.8	44.3
Issa	leaves	*Uapaca*	*kirkiana*	1.7	-28.0	1.2	44.9
Issa	leaves	*Vitex*	*doniana*	0.9	-28.1	1.0	48.0
Issa	leaves	*Julbernardia*	*globiflora*	2.2	-29.0	2.0	51.2
Issa	leaves	*Parinari*	*curatellifolia*	1.5	-30.5	1.3	50.2
Issa	leaves	*Brachystegia*	*microphylla*	1.4	-28.9	2.0	53.9
Issa	leaves	*Brachystegia*	*spiciformis*	1.2	-28.5	2.1	54.1
Issa	leaves	*Annona*	*senegalensis*	-1.2	-26.8	1.4	48.6
Issa	leaves	*Pterocarpus*	*tinctorius*	-1.1	-26.8	2.3	54.0
Issa	leaves	*Diplorhynchus*	*condylocarpon*	1.7	-25.7	1.3	52.4
Issa	leaves	*Uapaca*	*nitida*	0.5	-29.0	1.1	50.9
Issa	leaves	*Piliostigma*	*thonningii*	2.6	-27.4	1.5	45.5
Issa	grass	*inidentified*	*na*	3.4	-15.0	1.4	42.9

* single samples only
