# Supplementary material for: Food mechanical properties and isotopic signatures in forest versus savannah dwelling eastern chimpanzees
Source: Commun Biol. 2018 Aug 10;1:109. doi: 10.1038/s42003-018-0115-6 (PMC6123729; doi:10.1038/s42003-018-0115-6)
Supplement: Supplementary file 2 — Description of Additional Supplementary Information [file 42003_2018_115_MOESM2_ESM.docx]

**Description of Additional Supplementary Files**

File Name: Supplementary Movie 1

Description: A brief video illustrating the opening of a seed pod by a chimpanzee. Note the use of the anterior dentition for tackling the mechanically challenging exocarp
